# Microbial, Physicochemical, and Flavor Interactions in High-Temperature Sauce-Flavor *Daqu*

**DOI:** 10.3390/biology14101324

**Published:** 2025-09-25

**Authors:** Youwei Chen, Limei Zou, Luyao Wang, Weiwei Dong, Yanli Feng, Xiang Yu, Jun Liu, Yu Zhang, Yuanliang Hu, Shenxi Chen

**Affiliations:** 1 Hubei Key Laboratory of Edible Wild Plants Conservation and Utilization, College of Life Sciences, Hubei Normal University, Huangshi 435002, China; ywchen@hbnu.edu.cn (Y.C.); cyw13026303729@163.com (L.W.); weiweidong@hbnu.edu.cn (W.D.); fengyanli@hbnu.edu.cn (Y.F.); xiangyu@hbnu.edu.cn (X.Y.); jliu@hbnu.edu.cn (J.L.); zhangyu@hbnu.edu.cn (Y.Z.); 2 Hubei Provincial Key Laboratory of Occurrence and Intervention of Kidney Diseases, Hubei Provincial Engineering Research Center of Immunotherapy Drugs for Renal Tumors, Hubei Polytechnic University, Huangshi 435003, China; 203166@hbpu.edu.cn; 3 Hubei Key Laboratory of Quality and Safety of Traditional Chinese Medicine Health Food, Jing Brand Co., Ltd., Daye 435199, China

**Keywords:** sauce-flavor *Daqu*, high-temperature fermentation, microbial succession, physicochemical dynamics, flavor compounds

## Abstract

Sauce-flavor Daqu is the solid-state fermentation starter for sauce-flavor Baijiu. Its microbial community influences flavor formation, yet links between community change, process conditions, and flavor development during high-temperature fermentation remain unclear. We investigated Daqu fermentation by integrating high-throughput sequencing, monitoring of physicochemical parameters, and analysis of volatile compounds. Fermentation temperature showed three phases: rapid rise, fluctuating plateau, and gradual decline. High temperatures were associated with increased thermophilic microbes such as *Bacillus* and *Thermoascus* and with higher levels of reducing sugars and amino acid nitrogen; amylase, protease, and other hydrolase activities were detected. Bacterial composition varied more than fungal composition; *Firmicutes* and *Ascomycota* were the dominant phyla, and *Bacillus* and *Thermoascus* were abundant genera. Canonical correspondence analysis associated reducing sugars, acidity, and moisture with early community shifts, and amino acid nitrogen with later shifts; reducing sugars and moisture showed the strongest associations. Filamentous fungi and *Bacillus* correlated with pyrazine-type compounds. These results link microbial composition, process parameters, and flavor profiles, and may inform the standardization and mechanization of Daqu production.

## Simple Summary

Sauce-flavor Daqu is the traditional starter used to make a type of Chinese liquor with a characteristic aroma. It is produced by mixing grains and allowing naturally occurring microbes to grow in a warm, open process. How these microbes and the changing conditions shape flavor is not fully understood. In this study, we tracked temperature, moisture, acidity, and sugars over a 52-day fermentation while recording which microbes were present and which aroma compounds formed. Temperature followed three stages—rise, plateau with fluctuations, and decline—which coincided with an increase in heat-tolerant bacteria and molds. These organisms help break down starch and protein into smaller molecules that can become flavor compounds. Some groups were closely linked to nut-like aroma substances called pyrazines. Our results clarify how process conditions relate to microbes and flavor, and they may help producers improve quality and consistency while preserving traditional practice.

## 1. Introduction

Sauce-flavor Baijiu, known for its distinctive flavor profile and superior quality, is widely favored by consumers and is regarded as one of the six major categories of distilled spirits globally [1]. Compared to traditional strong-aroma Baijiu, which is produced using medium-temperature *Daqu*, and light-aroma Baijiu, which employs low-temperature *Daqu*, sauce-flavor Baijiu is distinguished by the use of high-temperature *Daqu*, with fermentation temperatures exceeding 50 °C and sustained for over 50 days [2,3,4].

These differences in fermentation temperature and process duration led to distinct microbial community structures and flavor formation pathways. For example, strong- and light-aroma *Daqu* are typically dominated by mesophilic genera such as *Lactobacillus*, *Aspergillus*, and *Candida*, which contribute primarily to ester-type aroma compounds [2,4]. In contrast, sauce-flavor *Daqu* supports the enrichment of thermophilic microorganisms such as *Bacillus* and *Thermoascus*, which are associated with the production of pyrazines, acids, and other heat-derived volatiles [4]. The current study further highlights these distinctions by linking stage-specific microbial succession and physicochemical changes to the formation of sauce-flavor-specific aroma compounds, thereby providing a comparative perspective on how fermentation parameters shape the microbial ecology and flavor profiles of different Baijiu types.

*Daqu* is a traditional fermentation starter essential for producing sauce-flavor Baijiu. It is made from wheat, barley, and peas, which are mixed, inoculated with previously fermented mature *Daqu* (serving as inoculum *Daqu*), molded into bricks, and fermented under open, high-temperature, solid-state conditions for about 52 days [5]. This unique process creates a selective environment for thermophilic microorganisms such as *Bacillus* and *Thermoascus*, whose metabolic activities play a direct role in the formation of the distinctive sauce-flavor compounds [5]. The production of sauce-flavor *Daqu*, a critical raw material, is a complex process strongly influenced by geographic and environmental factors, including seasonal variation. These influences lead to considerable variability in *Daqu* quality, which ultimately affects the flavor consistency and stability of the final Baijiu product [6]. Traditionally, the *Daqu* production process has relied heavily on the experience of individual workers, leading to quality inconsistency and highlighting the urgent need for technological intervention and process standardization [7]. The study of the key quality features of sauce-flavor *Daqu* and their influencing factors is essential for improving quality control and promoting the industrial development of sauce-flavor Baijiu.

Recent studies have demonstrated that the quality of sauce-flavor *Daqu* is driven by three core factors: (i) environmental microbial communities, such as thermotolerant fungi like *Thermoascus*, (ii) raw material metabolism, for example, the degree of wheat starch gelatinization, and (iii) dynamic physicochemical parameters including the duration of high-temperature stages [1,8,9]. However, most existing studies are limited to single factor analyses and lack spatiotemporal resolution of the cascading effects among temperature, microbial metabolism, and the transformation of flavor precursors. For instance, it remains unclear how proteases secreted by microbes during high-temperature phases regulate the relationship between amino acid nitrogen and pyrazine compounds [10,11]. While traditional cultivation techniques can identify microbial species, they are inefficient and insufficient for elucidating the overall community structure. In contrast, high-throughput sequencing effectively reveals microbial diversity, yet its integration with physicochemical parameters and flavor compounds remains underexplored [12,13]. Furthermore, significant variations exist in the microbial communities and flavor profiles of *Daqu* across different regions, seasons, and environmental conditions [14,15]. Current research predominantly focuses on single-variable factors, with a notable lack of comprehensive, dynamic, and multi-factorial association studies. Accordingly, this study aims to explore how the spatiotemporal dynamics of microbial communities under high-temperature fermentation conditions influence key enzymatic processes and flavor precursor transformation in sauce-flavor *Daqu*.

This study focuses on sauce-flavor *Daqu* traditionally produced in Maotai Town, Guizhou Province. Through comprehensive spatiotemporal sampling, we systematically investigate the dynamic changes in key physicochemical parameters during fermentation, including temperature, moisture content, acidity, and reducing sugars. Flavor compounds are quantitatively analyzed using headspace solid-phase microextraction coupled with gas chromatography–mass spectrometry (HS-SPME-GC-MS). In parallel, both traditional culturing methods and high-throughput sequencing are employed to characterize the structure and dynamic succession of the microbial community. By integrating multivariate statistical analyses, we elucidate the causal relationships between microbial functional differentiation under high-temperature selection pressure and flavor compound formation. This approach enables the construction of a theoretical framework for fermentation regulation based on multi-factor associations and the identification of key functional microbial taxa. Our findings provide a scientific basis for quality control and standardized production of sauce-flavor *Daqu*, offering valuable insights for the development of mechanized *Daqu* manufacturing processes.

## 2. Materials and Methods

### 2.1. Sample Collection

The *Daqu* samples used in this study were collected from a traditional distillery located in Moutai Town, Guangdong Province, China. All samples were produced by a 52-day open, high-temperature, solid-state heap fermentation in a ventilated Daqu room, where coarsely milled wheat was back-slopped with mature Daqu, pressed into bricks and stacked as free-standing heaps that self-heated (>50 °C). Samples were collected from a single production batch at four key time points: Day 0 (initial stage), Day 10 (first turning), Day 21 (second turning), and Day 52 (final stage). At each time point and location, samples were collected in triplicate to ensure reproducibility.

To account for spatial heterogeneity within the fermentation chamber—where preliminary trials revealed temperature and humidity differences of up to 8–12 °C between layers nine sampling sites were established across three vertical layers of the fermentation stack: upper (H1, H4, H6), middle (M1, M4, M6), and lower (L1, L4, L6) (see Figure S1).

Each sample was divided into two portions: one was immediately stored at 4 °C for physicochemical analysis and culturable microbial isolation, and the other was frozen at −80 °C for flavor compound analysis and high-throughput sequencing.

### 2.2. Physicochemical Analyses

Button-type temperature loggers were embedded in the *Daqu* bricks during pressing, with temperature data recorded every two hours using Obutton64 software (Version 2.0; Omega Engineering Inc., Norwalk, Connecticut, USA)

Analyses of moisture, acidity, reducing sugars, fermentation capacity, and amino acid nitrogen were performed in accordance with the Chinese industry standard QB/T 4257-2011 *General Analytical Methods for Brewing Daqu* [16]. Moisture content was determined by oven-drying samples at 105 °C to constant weight and expressed as a percentage of the original sample mass. Acidity was determined by acid-base titration using 0.1 mol/L NaOH standard solution, with the endpoint set at pH 8.2. The results were expressed in millimoles per 10 g of sample. Reducing sugars were quantified using the Fehling reaction, with a 2 g/L glucose standard solution for quantification. Results were expressed as a mass percentage of the sample [17]. Fermentation capacity was assessed by measuring the mass of CO_2_ produced. Specifically, sorghum juice with a concentration of 7°Bé was fermented for 72 h, and the generated CO_2_ was collected and weighed. The results were expressed as grams of CO_2_ per 0.5 g of *Daqu* over 72 h. Amino acid nitrogen was determined by formaldehyde titration. Briefly, 20 g of *Daqu* was soaked in water, filtered, and 20 mL of the filtrate was titrated with 0.05 mol/L NaOH to pH 8.2. After adding 10 mL formaldehyde, titration continued to pH 9.2. A blank with distilled water was used, and results were expressed in g/kg [18]. Neutral protease activity was determined according to the industry standard SB/T 10317-1999 [19]. Briefly, 5.0 g of *Daqu* sample was extracted with 50 mL of 100 mM phosphate buffer (pH 7.5) at 40 °C for 30 min in a water bath, and the filtrate was used as the crude enzyme solution. For the assay, 1 mL of enzyme solution was preheated at 40 °C for 5 min, followed by the addition of 1 mL of 2% casein solution. After incubation at 40 °C for 20 min, the reaction was terminated by adding 2 mL of 0.4 mol/L trichloroacetic acid (TCA). The mixture was left to stand for 10 min and then centrifuged at 8000 rpm for 30 s. Then, 1 mL of supernatant was mixed with 5 mL of 0.4 mol/L sodium carbonate solution and 1 mL of Folin reagent. The mixture was incubated at 40 °C for 20 min, and absorbance was measured at 680 nm. A blank control was prepared by adding TCA before the casein substrate. α-Amylase activity was determined via the iodine–starch colorimetric method. Enzymes were extracted with 100 mM phosphate buffer (pH 6.0), and starch was used as the substrate. Absorbance was measured at 620 nm. Glucoamylase activity was measured using the DNS method with 2% soluble starch as the substrate. Briefly, 5.0 g of *Daqu* was extracted with 54 mL distilled water and 6 mL acetate buffer at 35 °C for 1 h, and the filtrate was used as crude enzyme. For the assay, 5 mL starch solution was preheated at 35 °C for 3–5 min, then 0.5 mL enzyme was added and incubated for 20 min. After adding 1.5 mL DNS, the mixture was boiled for 15 min, cooled, diluted with 10.5 mL water, and absorbance was measured at 540 nm [20].These measurements cover key fermentation pathways including saccharification (reducing sugars), proteolysis (amino acid nitrogen), and acid metabolism (acidity), all of which are directly related to the formation of flavor precursors.

### 2.3. Flavor Compound Analysis

#### 2.3.1. Semi-Quantitative Analysis

Approximately 2.0 g of *Daqu* was placed in a 20 mL headspace vial. Subsequently, 3.0 g NaCl, 10 mL of 10% ethanol solution, and 20 μL of the internal standard mixture (ethyl hexanoate, 1-hexanol, ethyl octanoate, and hexanal) were added. After vortexing for 5 min, samples were extracted using HS-SPME and analyzed by GC-MS equipped with a DB-FFAP column (60 m × 0.25 mm × 0.25 μm). The GC oven temperature was programmed from 40 °C (hold 2 min) to 250 °C at a rate of 3.5 °C/min. Mass spectrometry was operated in electron impact (EI) mode at 70 eV. The scan range was set from *m*/*z* 20 to 500. Compounds with a library match similarity of ≥75% were semi-quantified using the internal standard method under selected ion monitoring (SIM) mode [20].

#### 2.3.2. Quantitative Analysis

Quantification was performed according to the Methods for Baijiu Analysis (GB/T 10345-2007) [21]. A 5.0 g sample was extracted with 15 mL of 10% ethanol, then subjected to ultrasonicated in an ice bath for 30 min, and stored overnight at 4 °C. After centrifugation at 8000 rpm for 5 min, 1 mL of the supernatant was spiked with 10 μL of the internal standards, including tert-pentanol, n-pentyl acetate, 2-ethylhexanol. GC-FID analysis was performed using nitrogen as the carrier gas and a temperature program optimized for ester detection [20].

This dual-method approach was selected to ensure both qualitative comprehensiveness and quantitative reliability. HS-SPME-GC-MS enables sensitive detection of trace volatiles, while GC-FID ensures accurate quantification of major esters. The internal standards were selected to represent key aroma compound classes and retention times, thereby enhancing method robustness and comparability.

### 2.4. Enumeration of Culturable Microorganisms

A 10.0 g sample of *Daqu* was homogenized with 90 mL of sterile distilled water and then shaken for 30 min, followed by serial tenfold dilutions. Lactic acid bacteria were enumerated using the pour plate method by inoculating the dilutions (10^−3^ to 10^−6^) onto MRS agar (Oxoid) and incubating at 37 °C for 48 h. *Bacillus*, lactic acid bacteria (LABs), yeasts, and molds were enumerated using the spread plate method. *Bacillus* and LABs were plated on nutrient agar (NA, Difco) and incubated at 37 °C for 24 h. Yeasts and molds were plated on Bengal red agar (HKM) and incubated at 30 °C for 48 h [20].

### 2.5. Identification of Culturable Microorganisms

Representative single colonies were picked from enumeration plates for subculturing. Bacteria and lactic acid bacteria were inoculated onto nutrient agar and MRS agar plates, respectively, and incubated at 37 °C for 24–48 h. Yeasts and molds were cultured on potato dextrose agar at 30 °C for 48 h. DNA was extracted from pure cultures, and PCR amplification was performed using a Bio-Rad T100 thermal cycler. For bacteria and lactic acid bacteria, single colonies were lysed in 80 μL lysis buffer (95 °C for 30 min, 4 °C for 10 min). The PCR mixture (30 μL) contained 12 μL rTaq mix, 2 μL lysate, 15 μL ultrapure water, and 0.5 μL each of primers 27F and 1429R. The PCR program was: 94 °C for 5 min; 35 cycles of 94 °C for 30 s, 52 °C for 50 s, 72 °C for 1 min; final extension at 72 °C for 10 min. For fungi (molds and yeasts), DNA was extracted using a commercial kit, and PCR was conducted with primers ITS1 and ITS4 under the same system, but with 30 cycles and annealing at 55 °C. The quality and quantity of the extracted genomic DNA (gDNA) were assessed using a NanoDrop spectrophotometer (Thermo Fisher Scientific, Waltham, Massachusetts, USA). DNA purity was evaluated by measuring the A260/A280 ratio, and DNA concentration was determined based on absorbance at 260 nm.

### 2.6. Microbial Community Analysis by High-Throughput Sequencing

Genomic DNA was extracted from *Daqu* samples using a commercial kit (OMEGA D3350 and D3390, OMEGA Bio-Tek, Newark, DE, USA) following the manufacturer’s instructions. Negative controls were included during DNA extraction and PCR amplification to monitor potential contamination. The bacterial 16S rRNA gene was amplified using primers 27F (AGRGTTTGATYNTGGCTCAG) and 1492R (TASGGHTACCTTGT-TASGACTT), while the fungal ITS region was amplified with ITS1-F (CTTGGTCATTTAGAGGAAGTAA) and ITS4 (TCCTCCGCTTATTGATATGC) [22,23]. Amplicons were sequenced on an Illumina HiSeq 2500 platform (Illumina, Inc., San Diego, CA, USA) using a paired-end sequencing protocol with 250 base pairs (bp) per read (i.e., PE250).

Raw sequences were quality-filtered and merged using USEARCH, and operational taxonomic units (OTUs) were clustered at 97% similarity. Although amplicon sequence variant (ASV) methods are increasingly used, we adopted the OTU-based pipeline to facilitate comparison with previous studies on *Daqu* microbiota.

Prior to diversity analysis, all samples were rarefied to the minimum sequencing depth to standardize read counts and reduce bias. Alpha diversity indices (Shannon, Simpson, Chao1, ACE) were calculated based on the rarefied OTU matrix using BIO-DAP software (Fundy National Park, Moncton, NB, Canada). Bray–Curtis dissimilarity was used to calculate beta diversity, followed by principal coordinate analysis (PCoA) and PERMANOVA (Adonis test, 999 permutations) for group significance testing. Taxonomic profiles and PCA plots were visualized using Origin 2020.

### 2.7. Data Analysis and Visualization

The raw data generated from high-throughput sequencing (HTS) has been securely archived in NCBI under the BioProject accession number PRJNA1293917 and PRJNA1293933. Experimental data were recorded using Microsoft Office 2019. Statistical analyses were performed using IBM SPSS Statistics 25. One-way analysis of variance (ANOVA) was conducted to compare differences among groups, followed by Tukey’s post hoc test to assess pairwise significance. Prior to ANOVA, normality and homogeneity of variance were evaluated using Shapiro–Wilk and Levene’s tests, respectively. A *p*-value of less than 0.05 was considered statistically significant. All experiments were conducted in triplicate (*n* = 3). Graphical visualizations were generated using Origin 2022b.

## 3. Results and Discussions

### 3.1. Dynamics of Cultivable Microbial Populations

Enumeration results (Table 1) revealed spatiotemporal variations in the abundance of culturable microbial communities during *Daqu* fermentation, suggesting intricate interactions among microbial taxa. *Bacillus* spp. consistently dominated (1.22 × 10^7^ to 9.71 × 10^8^ cfu/g), owing to their thermotolerance and desiccation resistance, which align with the high-temperature and low-moisture conditions of sauce-flavor *Daqu* fermentation [24]. Lactic acid bacteria (LABs, 3.28 × 10^6^ to 6.21 × 10^7^ cfu/g) proliferated during early-stage fermentation, likely suppressing undesirable microbes via acidification [25]. Their decline in later stages may be attributed to substrate depletion or the accumulation of excessive acid. Yeasts (0–1.83 × 10^6^ cfu/g) increased notably during the second turning stage, possibly benefiting from fermentable sugars released by mold-driven polysaccharide degradation. However, in LABs -enriched regions (e.g., L6), yeast growth was likely suppressed by low pH and nutrient competition. Mold counts increased gradually, peaking in the final stage (e.g., L1: 6.89 × 10^6^ cfu/g). Their acid tolerance and hydrolytic enzymes secretion (e.g., amylases and proteases) facilitate polymer degradation [26,27], which promotes their own expansion and indirectly supporting *Bacillus* proliferation Meanwhile resource competition may suppress other taxa, altering the community structure and metabolic balance. These observations underscore a dynamic microbial network shaped by environmental selection and metabolic interactions.

### 3.2. Physicochemical Changes During Fermentation

**Figure 1:**
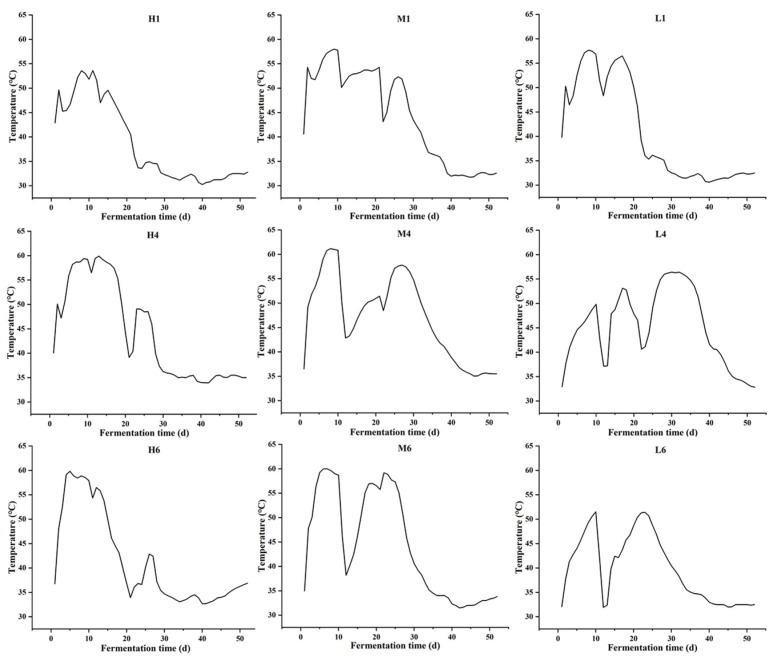
The temperature of *Daqu* at different positions during fermentation changed with fermentation time. Note: H1, M1, L1, H4, M4, L4, H6, M6, L6 correspond to the respective positions in Figure S1.

Temperature emerged as a key driver of microbial succession in sauce-flavor *Daqu* fermentation [28]. During the initial fermentation phase (0–10 days), the core temperature rapidly increased, reached peak values ranging from 51.5 °C to 61.2 °C. This high-temperature environment (>50 °C) led to selective enrichment of thermotolerant taxa such as *Bacillus* and specific LABs, which is consistent with the traditional “high-temperature purification” stage of *Daqu* production [29,30] (Figure 1). Following the turning operation, the temperature gradually declined, likely as a result of reduced moisture content and diminished metabolic activity of thermophilic microorganisms. In addition, manual interventions such as turning and aeration can induce temperature fluctuations, which subsequently influence microbial succession and metabolic dynamics [31]. Sustained high temperatures inhibited undesirable microbes, facilitating starch saccharification, and promoted the accumulation of flavor compounds [32].

**Figure 2:**
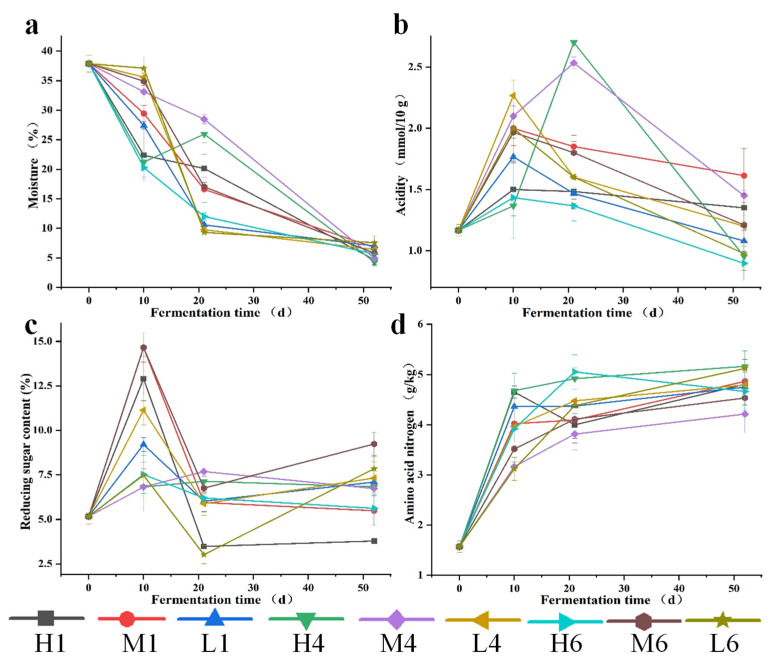
The physicochemical factors of three stages *Daqu* during fermentation, including moisture (**a**), acidity (**b**), reducing sugar (**c**), and amino acid nitrogen (**d**). Note: H1, M1, L1, H4, M4, L4, H6, M6, L6 correspond to the respective positions in Figure S1.

Moisture content decreased from an initial 37.8% to less than 10% by the end of fermentation, highlighting the progressive dehydration of *Daqu* (Figure 2a). Notably, upper layers showed a faster moisture loss due to evaporation. During the mid-fermentation phase, sharp moisture declines were observed at L4 and L6, coinciding with a significant increase in *Bacillus* populations (6.58 × 10^8^ cfu/g and 1.34 × 10^8^ cfu/g, respectively; Table 1). This spatial correlation suggests that microbial metabolism-along with the heat it generates-may accelerate localized dehydration. Toward the end of fermentation, moisture levels stabilized, likely due to reduced microbial activity and structural compaction of *Daqu* bricks.

Acidity exhibited a characteristic a “rise-then-fall” trend (Figure 2b), with peak values observed in most samples on Day 10. This increase was driven by LABs, which maintained metabolic activity under elevated temperatures and significantly acidified the environment during early to mid-fermentation stages. Notably, L4 exhibited the highest acidity (2.27 mM/10 g), which coincided with the peak LAB count (3.50 × 10^7^ cfu/g), suggesting that lactic acid production was particularly active in moist and thermally favorable conditions. In some mid-fermentation samples (e.g., H4 and M4), acidity continued to rise in parallel with sustained LAB growth (Table 1). These dynamics changes in acidity not only reflect LAB metabolic activity but also reshape the microbial microenvironment, thereby modulating microbial interactions and, in turn, altering the dominant metabolic pathways and flavor compound formation.

Similarly to acidity, reducing sugar content also followed a “rise-then-fall” trend (Figure 2c). In early fermentation, *thermophilic* *Bacillus* species (Table 1) secreted amylases that rapidly degraded starches into soluble sugars, contributing to the accumulation of reducing sugars. After Day 10, sugar levels declined sharply in most sampling regions, for example, reducing sugars at H1 dropped from ~12.5% to 2.5%, correlating with a marked increase in yeast abundance (from 1.94 × 10^4^ to 6.90 × 10^4^ cfu/g). This trend likely reflects the intensified glycolytic activity of yeasts, which rapidly consumed the available reducing sugars for energy and growth. Interestingly, a secondary increase in sugar levels was observed in some samples after Day 20, which appears counterintuitive as sugars are generally consumed during fermentation. This phenomenon may be attributed to the delayed activation of certain polysaccharide-degrading microorganisms, such as *Thermoascus* and *Aspergillus*, which become more dominant in the later stages under aerobic and high-temperature conditions [33,34]. Their secreted enzymes, particularly cellulases and hemicellulases—could hydrolyze complex structural polysaccharides into additional reducing sugars, partially replenishing sugar levels despite ongoing microbial consumption. In the final phase, sugar levels stabilized, likely because of carbon source depletion and reduced yeast metabolic activity due to environmental constraints. Overall, early sugar accumulation was driven by hydrolytic activities of molds and bacteria, while mid-to-late stages saw yeast-dominated sugar metabolism, providing essential energy and carbon for flavor precursor synthesis.

Amino nitrogen content continuously increased throughout fermentation (Figure 2d). During early fermentation, amino nitrogen levels rose rapidly in tandem with increasing *Bacillus* counts, which may indicate that *Bacillus*-driven enzymes activities degraded proteins. In the mid-phase, peripheral regions (e.g., H6 and L6) exhibited sharp increases in amino nitrogen, coinciding with localized spikes in *Bacillus* populations (Table 1), likely due to favorable microenvironmental conditions. Additionally, proteolytic activity may have been further enhanced by the synergistic action of mold-derived cellulases and proteases. The stabilization of amino nitrogen levels in the final stage may be attributed to substrate depletion or microbial assimilation. These dynamics underscore the metabolic cooperation among microbial taxa in nitrogen release and transformation, which are essential for the synthesis of key flavor precursors in sauce-flavor Baijiu.

### 3.3. Enzyme Activities and Fermentation Capacity

**Figure 3:**
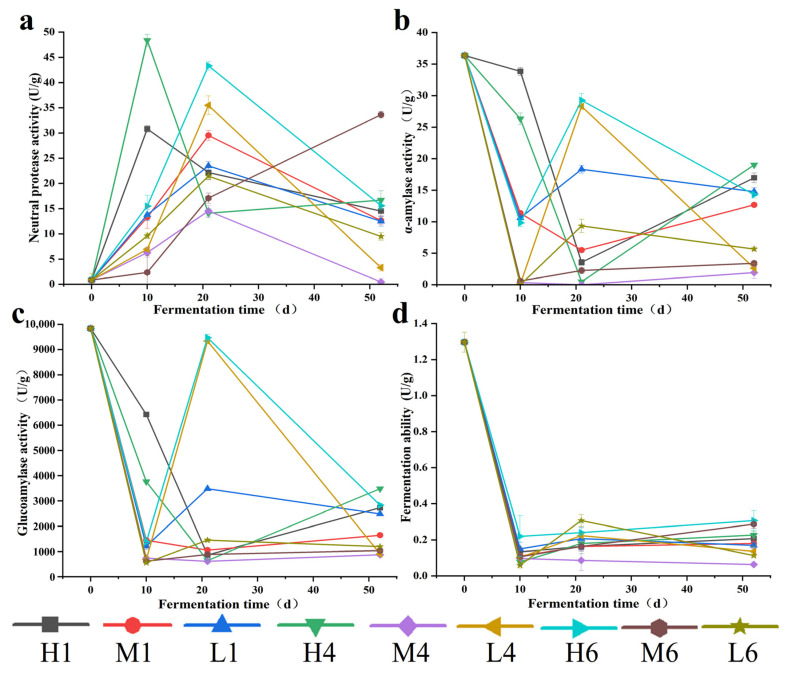
The fermenting vitalities of three stages *Daqu* during fermentation, including neutral protease (**a**), α-amylase (**b**), glucoamylase activity (**c**), and fermenting ability (**d**). Note: H1, M1, L1, H4, M4, L4, H6, M6, L6 correspond to the respective positions in Figure S1.

Enzyme activity during *Daqu* fermentation exhibited pronounced spatial and temporal heterogeneity (Figure 3). In the initial phase (0–10 days), protease activity peaked at sites H4 and H1 (48.37 U/g and 30.82 U/g, respectively), likely driven by rapid proliferation of *Bacillus* and its adaptation to thermophilic conditions (Table 1). Similar findings have been reported in sauce-flavor *Daqu*, *Bacillus*-secreted proteases hydrolyzed proteins to release peptides and free amino acids, providing precursors for the biosynthesis of pyrazines and other flavor compounds [34]. In the post-first-turning phase (10–21 days), protease activity rebounded at L4 and H6, likely due to improved aeration and secondary proliferation of *Bacillus* (Table 1). During the late fermentation phase, protease activity gradually declined, likely due to substrate exhaustion and reduced microbial metabolism.

α-Amylase and glucoamylase activities followed similar trends (Figure 3b,c), and were primarily attributed to molds (e.g., *Aspergillus*, *Rhizopus*) and yeasts. In early fermentation, enzyme activity remained low at central sites (L4, M4, M6) due to elevated temperatures and hypoxia, which inhibited fungal and yeast growth. After turning, the combined effect of lower temperatures and increased aeration facilitated fungal growth, in line with observations reported in high-temperature *Daqu* [35], thereby contributing to elevated enzyme activities at sites L4 and H6, where fungal abundance was higher (Table 1). In the later stage, enzyme activities showed a further increase in localized regions such as M6 and L6, possibly due to localized expansion of enzyme-producing fungi, indicating their sustained contribution to starch degradation.

Fermentation power (Figure 3d), representing the yeast-driven ethanol production potential, showed a decline after initial high values (~1.3 U/g), likely due to thermal suppression of yeast activity. This aligns with prior studies reporting yeast growth inhibition under heat stress in high-temperature *Daqu* [36]. A partial recovery post-second turning, especially in L4, indicates that cooler temperatures and improved aeration transiently restored yeast metabolism. Similar recovery trends have been noted in other *Daqu* systems following pile reshaping [37].

In summary, the dynamic changes in protease, α-amylase, and glucoamylase activities were governed by the spatial distribution and environmental adaptability of enzyme-producing microorganisms as well as fermentation management practices. Fermentation power was primarily driven by yeast activity and served as an indicator of the temperature-mediated regulation of ethanol production potential. Together, these enzymatic and metabolic dynamics coordinated the supply and transformation efficiency of flavor precursors within the fermentation system.

### 3.4. Dynamics of Flavor Compounds

**Figure 4:**
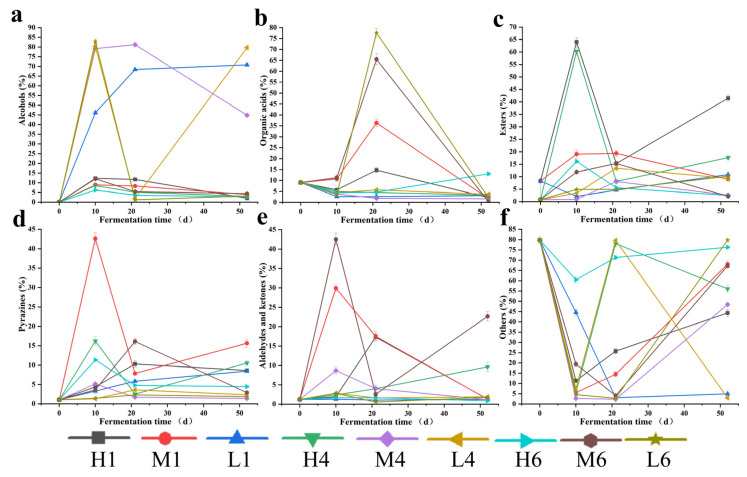
The variation in main flavor compounds in three stages of *Daqu* during fermentation, including alcohols (**a**), acids (**b**), esters (**c**), pyrazines (**d**), aldehydes and ketones (**e**), and the others (**f**). Note: H1, M1, L1, H4, M4, L4, H6, M6, L6 correspond to the respective positions in Figure S1.

The formation and transformation of flavor precursors directly determine the aroma characteristics and style differentiation of the final Baijiu product [38]. In this study, six representative classes of flavor compounds—including alcohols, organic acids, esters, pyrazines, aldehydes and ketones, and others—were profiled for their spatiotemporal dynamics to elucidate their evolution patterns and potential microbial mechanisms across different fermentation stages and locations (Figure 4).

Alcohol levels generally followed an increase–then–decrease pattern (Figure 4a), consistent with previous findings [39]. For instance, in sample H1, the alcohol concentration rose from 0.07% at the start of fermentation to a peak of 12.30%, then declined to 2.00% in the late stage. As key intermediate metabolites formed during mid-fermentation, alcohols can be further transformed into esters or aldehydes, serving as carbon sources and precursors for the synthesis of aroma-active compounds. Interestingly, in some samples, alcohol levels remained stable or even increased in the late stage, suggesting ongoing microbial activity. This phenomenon may be explained by the continued metabolic activity of yeasts such as *Saccharomyces* and *Wickerhamomyces*, which, as previously reported [40], can utilize residual sugars and amino acids via the Ehrlich pathway to synthesize higher alcohols under aerobic conditions during the late fermentation stage. These alcohols may accumulate in surface-exposed regions of the fermentation matrix, contributing to late-stage flavor development.

Organic acids displayed a relatively moderate decreasing trend in some samples (Figure 4b). In specific regions such as L6, the organic acid content showed a sharp increase during the turning phase (from 3.56% to 77%), primarily due to enhanced activity of lactic acid bacteria. However, in the later stage, the organic acid content declined, which was likely due to esterification or microbial utilization, as previously reported [41]. This process reflects a “metabolite retention effect,” in which the lactic acid produced by LABs during the early stage of fermentation undergoes sustained esterification to form ethyl lactate. As a result, the acidity peak appears later than the peak in bacterial abundance. This illustrates the temporal-spatial mismatch between microbial metabolism and flavor transformation.

Esters showed pronounced fluctuations (Figure 4c). At location H1, the ester content rose sharply from 8% after the first turning to 64.04%, before decreasing to 19.25% after the second turning. The observed pattern closely matched the fungal abundance at the same site (Table 1), supporting previous findings that filamentous fungi are key contributors to ester production in *Daqu* [42]. Notably, despite a significant decline in LAB populations during the late fermentation stage (Table 1), lactic acid produced earlier may continue to undergo esterification into ethyl lactate. This could explain the secondary increase in ester content observed at certain positions (e.g., H4) (Figure 4c), reflecting the unique “metabolite retention effect” characteristic of *Daqu* fermentation. Given that esters are key aroma compounds in Baijiu, their dynamic synthesis and degradation directly contribute to the complexity and richness of the final aroma profile.

Pyrazines, the hallmark aroma compounds of sauce-flavor Baijiu, contribute nutty, roasted, and toasty notes [43]. In the mid-layer (e.g., M1), pyrazine concentrations peaked during the first turning (41.65%) (Figure 4d), likely driven by increased abundance and metabolic activity of *Bacillus* and *Actinomycetes*, as previously reported by Dong et al. [41]. The microenvironmental conditions—moderate temperature and adequate ventilation—selectively enriched pyrazine-producing microorganisms, demonstrating the critical role of spatial heterogeneity in shaping signature flavor development.

Aldehydes and ketones exhibited marked spatial heterogeneity during fermentation (Figure 4e). For instance, in M6, their concentrations followed a “rise–fall–rise” pattern that paralleled the abundance trend of LABs (Table 1), suggesting a potential link to lactic acid bacterial metabolism in this region. The late stage increase in aldehydes may be attributed to the oxidative conversion of alcohols by microbial oxidoreductases, particularly under aerobic conditions near the surface of the fermentation matrix.

“Other flavor compounds,” mainly comprising aromatic and heterocyclic compounds (Figure 4f), showed notable dynamic variations. For instance, in sample L1, their content declined from 44.54% to 3.10%, illustrating the multidirectional nature of substrate degradation and product biosynthesis during fermentation, which contributes to the complexity of the flavor profile. These fluctuations may result from the shifting balance between compound production and degradation by different microbial groups. For example, certain bacteria and filamentous fungi may produce aromatic compounds during active metabolism, while others, including oxidative yeasts or aerobic bacteria, may further metabolize or volatilize them at later stages. Environmental changes such as temperature, oxygen availability, and substrate depletion likely further modulate this interplay, leading to sample-specific trajectories.

In summary, flavor compounds during *Daqu* fermentation exhibit significant spatiotemporal variations, shaped by the synergies between the microbial metabolism and its growing environment. The interconversion among alcohols, organic acids, and esters; the spatial accumulation of pyrazines and aldehydes/ketones; and fluctuations in minor compounds collectively shape the complex, multi-dimensional aroma profile characteristic of sauce-flavor Baijiu.

### 3.5. Microbial Community Structure and Diversity

#### 3.5.1. Alpha and Beta Diversity Analysis

Based on α- and β-diversity analyses, bacterial and fungal communities exhibited markedly different successional patterns during *Daqu* fermentation.

Results from the Chao1 and Shannon indices indicated that both bacterial and fungal communities exhibited the highest richness (Chao1) and diversity (Shannon) in the early stage of fermentation (Day 10), accompanied by substantial structural fluctuations. This suggests a phase of rapid microbial colonization and community reshaping, consistent with patterns observed in other studies on high-temperature *Daqu* fermentation [41].

In the mid-stage (Day 21), diversity declined significantly, likely due to intensified environmental selection pressures and dominance of specific taxa. This decline may be attributed to thermophilic taxa such as *Bacillus* and *Thermoascus*, which dominated the mid-stage due to their heat tolerance and strong enzymatic activity. These microbes can alter the microenvironment and outcompete others by rapidly consuming nutrients or producing antimicrobial compounds, potentially suppressing or excluding less adapted species.

By the late stage (Day 52), diversity partially recovered, and community structure tended to stabilize (Table 2), possibly indicating the establishment of a more resilient and functionally specialized microbial consortium. This recovery phase mirrors observations in other traditional Chinese fermentation systems, where community succession leads to ecological stabilization and functional optimization [2].

Bacterial communities displayed significant β-diversity differences across fermentation stages, as reflected by the dispersed distribution patterns among samples, indicating pronounced structural reassembly over time. In contrast, fungal communities were more tightly clustered and exhibited greater similarity, suggesting a relatively stable community composition throughout the fermentation process (Figure S2). These results are consistent with patterns observed in other studies on high-temperature *Daqu* fermentation [41].

In summary, microbial diversity shifts during *Daqu* fermentation were predominantly associated with bacterial communities, whose structures were more sensitive to fermentation stage transitions. Fungal communities, by contrast, demonstrated greater ecological stability. This divergent successional pattern provides both theoretical insights and empirical evidence for optimizing fermentation strategies and regulating key microbial consortia.

#### 3.5.2. Phylum-Level and Genus-Level Community Composition

To elucidate the dynamic shifts in microbial communities and their roles in flavor formation during *Daqu* fermentation, this study systematically analyzed the bacterial and fungal community structures at both the phylum and genus levels.

**Figure 5:**
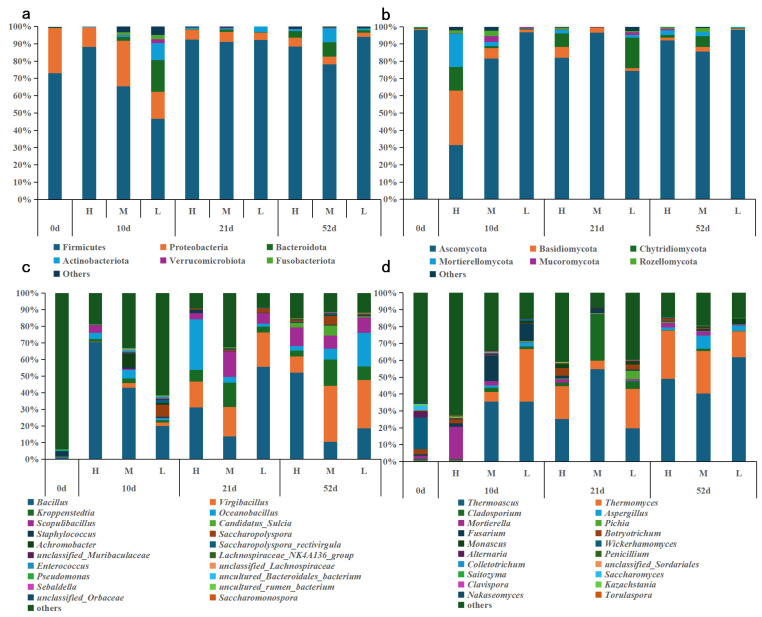
The microbial community of three stages of *Daqu* during fermentation, including bacterial phylum level (**a**), fungal phylum level (**b**), bacterial genus level (**c**), and fungal genus level (**d**).

At the phylum level, bacterial communities were primarily composed of *Firmicutes*, *Proteobacteria*, *Bacteroidota*, *Actinobacteriota*, *Verrucomicrobiota*, and *Fusobacteriota* (Figure 5a). Among these, *Firmicutes* consistently dominated throughout the fermentation process, in agreement with previous findings [44]. The relative abundance of *Firmicutes* exhibited distinct spatiotemporal dynamics. *Firmicutes* accounted for 73% of the raw *Daqu*. During fermentation, their abundance followed a “rise–fall” pattern in the upper (88% → 92% → 88%) and middle layers (64% → 91% → 78%), while continuously increasing in the lower layer (47% → 92% → 94%). *Firmicutes* are core functional taxa in fermentation, contributing to saccharification, flavor compound synthesis, and microecological stability [41]. Notably, *Proteobacteria* and *Actinobacteriota* were enriched in the lower layer during early fermentation, suggesting their potential role in environmental adaptation and metabolic transformation at this stage.

The fungal community was dominated by *Ascomycota*, *Basidiomycota*, *Chytridiomycota*, *Mortierellomycota*, *Mucoromycota*, and *Rozellomycota* (Figure 5b), with *Ascomycota* being overwhelmingly predominant, exhibiting significant spatial differentiation and dynamic variation. During the initial warehousing stage, *Ascomycota* accounted for 98%. Over the course of fermentation, their abundance increased steadily in the upper layer (31% → 82% → 92%), followed “rise–fall” trend in the middle layer (82% → 97% → 86%), and showed a “decline–recovery” pattern in the lower layer (97% → 74% → 98%). *Ascomycota* are well-adapted to high-temperature and low-moisture environments and play key roles in saccharification, flavor compound biosynthesis, and fermentation modulation, making them the core fungal group in *Daqu* fermentation [41].

At the genus level, the ten most abundant bacterial genera were *Bacillus*, *Virgibacillus*, *Oceanobacillus*, *Kroppenstedtia*, *Scopulibacillus*, *Weissella*, *Pediococcus*, *Enterobacter*, *Pantoea*, and *Staphylococcus* (Figure 5c). In the initial warehousing stage, these genera exhibited low relative abundance without clear dominance. However, by Day 10 of fermentation, *Bacillus* had emerged as the dominant genus across all layers, accounting for 70%, 43%, and 20% in the upper, middle, and lower layers, respectively. This dominance is attributed to the thermotolerance of *Bacillus* and its ability to produce lactic acid, which inhibits competing microbes. Notably, its abundance was highest in the upper layer, where the strongest α-amylase and neutral protease activities were also observed (Figure 3), consistent with the genus’s known capacity for hydrolase secretion [34].

Overall, *Bacillus* remained the most abundant genus across all fermentation stages and layers. Its thermotolerance and enzymatic activities (e.g., amylase and protease) underscore its central role in shaping the fermentation microecosystem, driving raw material degradation, and facilitating flavor compound generation, thereby contributing to fermentation quality and process stability. *Virgibacillus* and *Oceanobacillus* also demonstrated relatively high abundance in the upper and lower layers. Specifically, *Virgibacillus* increased from 0% to 16% and then declined to 10% in upper layer, and from 2% to 21% to 29% in the lower layer. Similarly, *Oceanobacillus* rose from 4% to 30% before dropping to 3% in the upper layer, while in the lower layer, it increased gradually from 1% to 2% and finally to 20%. Both genera are known to produce proteases and amylases, contributing to protein and starch degradation. They also participate in the synthesis of amino acid-based flavor precursors, thereby enhancing the nutritional value and aromatic complexity of the fermented product [45,46]. In the middle layer, *Virgibacillus* (3% → 18% → 34%) and *Kroppenstedtia* (3% → 15% → 16%) also exhibited notable abundance during fermentation. *Kroppenstedtia*, a thermophilic genus, is closely associated with amino acid production in *Daqu*. It can degrade complex polysaccharides such as cellulose and produce organic acids like lactic acid, thereby shaping both the fermentation microecology and flavor development [47,48]. The protease-producing capacity of these four dominant genera further corroborates the observed upward trend in amino nitrogen content during fermentation (Figure 2d).

For fungi, the ten most abundant genera were *Thermoascus*, *Thermomyces*, *Cladosporium*, *Mortierella*, *Aspergillus*, *Fusarium*, *Pichia*, *Botryotrichum*, *Wickerhamomyces*, and *Candida* (Figure 5d). In the initial warehousing stage, *Wickerhamomyces* accounted for 19%, making it the initially dominant genus. However, its abundance dropped sharply after 10 days of fermentation, falling below 1% and remaining detectable only in the lower layer. This pattern indicates limited competitive persistence under high-temperature conditions. Among these, *Thermoascus* was consistently the dominant and most stable fungal genus across all layers. Its abundance increased significantly in the upper layer (from 0% in the mid stage to 49% by the end), the middle and lower layers exhibited a fluctuating trend (middle layer: 35%→55%→40%, lower layer: 35%→20%→62%). *Thermoascus* is a thermophilic fungus capable of secreting heat-resistant saccharolytic enzymes that facilitate starch degradation. It may also contribute to the formation of furan-based flavor precursors, making it one of the core functional fungi in *Daqu* fermentation [49,50]. *Thermomyces* also exhibited relatively high abundance across layers, with notable increase in the upper layer from mid-to-late fermentation (rising from 0% to 29%), a peak in the middle layer during the late stage (25%), and a gradual decline in the lower layer (from 31% to 15%). This genus produces lipases involved in lipid degradation, supporting ester flavor compound formation [51]. *Mortierella* was abundant in the early upper layer (19%) but declined rapidly to 2%. *Cladosporium* peaked at 27% in the middle layer during the mid-stage and declined to 1% by the end. *Fusarium* was primarily abundant in the early lower layer (10%), decreasing to 1% in the later stage. While these genres were not dominant at the end of fermentation, they may play auxiliary roles in flavor formation during specific stages. In conclusion, *Thermoascus* consistently maintained high abundance across all fermentation layers, serving as a dominant genus with robust environmental adaptability and key fermentative function. Its prevalence underscores its central role in shaping the fungal community structure and metabolic landscape of *Daqu* fermentation. *Candida* is presumed to contribute to ester formation in sauce-flavor *Daqu*, as it has been shown to correlate strongly with ester precursors in other *Daqu* types [52]. Its tolerance to dry, high-temperature conditions suggest it remains metabolically active in later fermentation stages [53]. Thus, *Candida* may enhance flavor complexity by participating in alcohol and ester biosynthesis under stress conditions [54].

#### 3.5.3. Canonical Correspondence Analysis (CCA) of Microbial–Environmental Interactions

**Figure 6:**
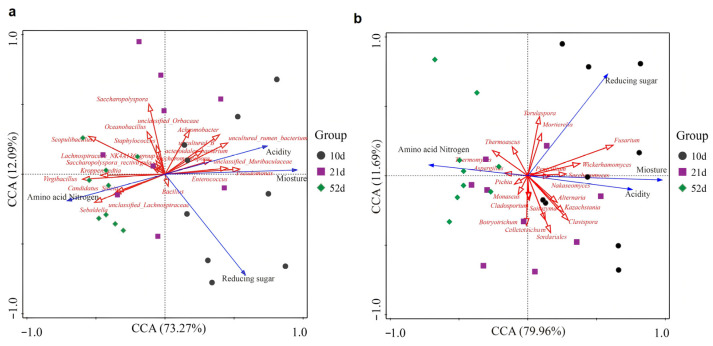
Canonical correlation analysis (CCA) between microbial communities and physicochemical parameters, based on the top 20 dominant genera. (**a**) Bacterial community; (**b**) fungal community. Each dot represents a sample, colored according to fermentation stage. Blue arrows indicate physicochemical variables, while red arrows represent core microbial genera. The length of each arrow reflects the strength of its influence.

To further elucidate the relationship between microbial community structure and key physicochemical factors during *Daqu* fermentation, canonical correspondence analysis (CCA) was conducted using the top 20 most abundant bacterial and fungal genera (Figure 6). For bacterial communities, the CCA model explained a cumulative 85.36% of the community variation (Figure 6a), while for fungal communities, CCA1 and CCA2 together accounted for 91.65% of the variation (Figure 6b), indicating a strong model fit and the capacity to effectively capture the associations between microbial structure and environmental variables.

In the CCA plots, the lengths of the environmental vectors reflect the relative strength of their influence on microbial community distribution. Among these factors, reducing sugars and moisture emerged as the primary drivers of microbial community dynamics, with acidity and amino acid nitrogen also significantly shaping community structure. Notably, amino acid nitrogen exerted an increasing influence during the later fermentation stages, underscoring its importance in protein hydrolysis and flavor compound biosynthesis. The sample distribution patterns further support this conclusion: early-stage samples clustered in the right quadrants and predominantly influenced by reducing sugars, moisture, and acidity; mid-stage samples were positioned near the origin, indicating a transitional microbial state; and late-stage samples shifted to the left quadrants, showing strong associations with amino acid nitrogen.

Among the dominant bacterial genera (Figure 6a), *Bacillus* exhibited positive correlations with reducing sugars, moisture, and amino acid nitrogen, but a negative correlation with acidity, highlighting its central role in carbohydrate metabolism and proteolysis. Previous studies have also confirmed the hydrolytic enzyme-producing capabilities of *Bacillus*, which is consistent with our findings [34,55]. Other genera including *Kroppenstedtia*, *Virgibacillus*, and *Oceanobacillus* showed positive correlations with amino acid nitrogen but negative associations with other environmental factors, suggesting their potential involvement in protein degradation and amino acid biosynthesis during the late fermentation stages. These genera have been reported to secrete proteases and amylases, facilitating substrate breakdown and the accumulation of flavor precursors [45,46,47,56], thus reinforcing their ecological functions.

For fungal genera, *Thermoascus* was positively correlated with both amino acid nitrogen and reducing sugars and negatively associated with moisture and acidity. This suggests a synergistic role in starch and protein hydrolysis. Previous studies have demonstrated that *Thermoascus* produces a range of amylases and proteases, contributing to efficient degradation of complex substrates [49,50]. *Thermomyces* also showed a positive correlation with amino acid nitrogen, alongside negative associations with reducing sugars, acidity, and moisture. Its strong proteolytic activity has been well documented [57]. In contrast, *Mortierella* and *Fusarium* were negatively correlated with amino acid nitrogen but positively associated with reducing sugars, acidity, and moisture. *Mortierella* is known to synthesize lactic acid and secrete α-glucosidase, while *Fusarium* produces amylases and organic acids. These metabolic traits support their involvements in carbohydrate metabolism and acidification during fermentation [58,59,60,61]. *Cladosporium* displayed negative correlations with amino acid nitrogen and reducing sugars, but positive correlations with acidity and moisture, reflecting its role in aromatic compound degradation and flavor biosynthesis. Additionally, generalist fungi such as *Pichia* and *Monascus* were mainly distributed near the center of the ordination plot, suggesting broad adaptability to various physicochemical conditions. *Pichia* is known for producing aroma-active esters and higher alcohols that enhance flavor complexity, as well as for its ability to degrade methanol, which contributes to product safety [62].

In summary, the CCA revealed a strong coupling between microbial community succession and environmental factors during *Daqu* fermentation. In the early stage, high levels of reducing sugars and moisture shaped the microbial structure by favoring the enrichment of amylolytic and lactic acid-producing microorganisms. The mid-stage was characterized by multifactorial regulation, while the late stage was strongly influenced by amino acid nitrogen accumulation, promoting the enrichment of proteolytic and flavor precursor-producing genera. These findings offer mechanistic insights into stage-specific environmental regulation of microbial succession, providing a theoretical basis for optimizing fermentation control strategies.

### 3.6. Correlation Between Microbial Taxa, Flavor Compounds, and Fermentation Performance

**Figure 7:**
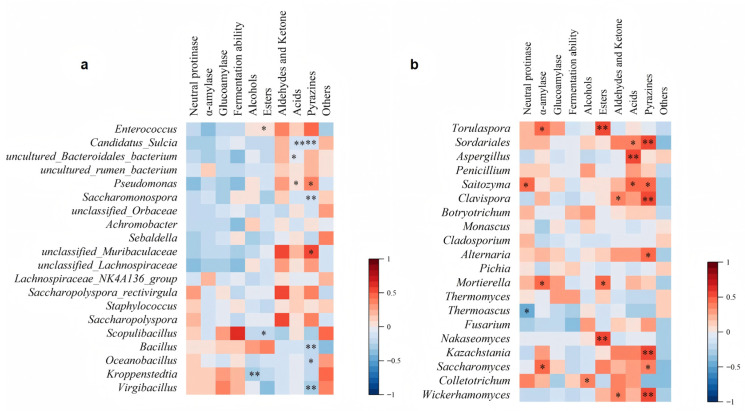
Heatmap showing the correlations between dominant microbial genera and flavor compounds or enzymatic activity. (**a**) Correlations with bacterial genera; (**b**) correlations with fungal genera. The top 20 dominant genera were selected for Spearman correlation analysis (* *p* < 0.05, ** *p* < 0.01), with an R-value threshold of 0.1.

During the maturation stage of *Daqu*, microbial communities metabolize substrates such as starch and proteins to synthesize a wide range of flavor compounds and functional enzymes. These metabolic products not only shape the characteristic aroma profile of Baijiu but also modulate biochemical processes throughout subsequent fermentation stages. To systematically elucidate how microbial taxa relates to flavor development, and fermentation performance, this study employed Spearman correlation analysis to characterize the relationships between dominant microbial genera, representative flavor components as well as major enzymatic activity parameters. The results presented in Figure 7 demonstrate the influence of core bacterial and fungal genera on flavor compound synthesis and fermentation vitality.

Among the dominant bacterial genera, *Enterococcus* showed a positive correlation with ester compounds. However, its specific metabolic role in ester formation during fermentation has not been fully elucidated and requires further investigation. By contrast, *Pseudomonas* and *Muribaculaceae* family exhibited statistically significant positive correlations with pyridine-type aroma compounds. Previous studies have indicated that *Pseudomonas* is capable of producing pyridine derivatives [63], whereas the low detection frequency of *Muribaculaceae* in Baijiu fermentation has limited research into its functional role.

In the fungal community, strong correlations between *Nakaseomyces* and *Torulaspora* with esters suggest their potential key roles in ester biosynthesis [42]. *Aspergillus* showed a significant positive correlation with organic acids, likely due to its capacity to secrete metabolites such as citric acid [64], which also contributed to increased acidity. Furthermore, *Sordariales*, *Clavispora*, *Kazachstania*, and *Wickerhamomyces* exhibited strong positive correlations with pyridine-type aroma compounds, indicating that these fungal taxa may also play important roles in the generation of pyridine-derived flavor components.

## 4. Conclusions

This study shows that the fermentation of sauce-flavor *Daqu* is a multi-stage system, characterized by concurrent changes in microbial communities and physicochemical factors. Sequential temperature variations were associated with microbial succession, particularly with the enrichment of thermotolerant taxa, including *Bacillus* and *Thermoascus*, which co-occurred with higher enzyme activities and increases in reducing sugars and amino acid nitrogen. Among physicochemical parameters, reducing sugars, acidity, and moisture were associated with community structure during the early phase, whereas amino acid nitrogen was more closely associated with later-stage shifts and with protein degradation and flavor-related metabolism.

Microbial communities varied over space and time, with bacterial dynamics being more variable than those of fungi. Throughout fermentation, *Bacillus* and *Thermoascus* were frequently abundant, consistent with their roles in flavor development. The production of pyrazine compounds was associated with *Bacillus* and certain molds, in line with observations from the volatile profiles.

This work describes links between microbes, environmental conditions, and metabolic products during sauce-flavor *Daqu* fermentation and may inform efforts toward process control and standardization. Future studies should test these associations with targeted functional measurements and controlled fermentations to evaluate causality.

## Figures and Tables

**Table 1:** The counts of cultivable microbes in different stages of *Daqu* samples during fermentation.

**Fermentation Days**	***Bacillus* (cfu/g)**								
	**H1**	**M1**	**L1**	**H4**	**M4**	**L4**	**H6**	**M6**	**L6**
**0d**	(3.85 ± 0.08) × 10^8^								
**10d**	(9.71 ± 0.20) × 10^8^	(9.42 ± 0.20) × 10^8^	(4.32 ± 0.08) × 10^8^	(5.97 ± 0.14) × 10^8^	(1.09 ± 0.07) × 10^8^	(4.32 ± 0.01) × 10^7^	(2.53 ± 0.08) × 10^8^	(1.22 ± 0.01) × 10^7^	(1.47 ± 0.01) × 10^7^
**21d**	(3.44 ± 0.08) × 10^8^	(5.04 ± 0.08) × 10^8^	(4.47 ± 0.16) × 10^8^	(2.85 ± 0.01) × 10^7^	(3.18 ± 0.15) × 10^8^	(6.58 ± 0.10) × 10^8^	(6.15 ± 0.12) × 10^8^	(2.52 ± 0.01) × 10^8^	(1.34 ± 0.01) × 10^8^
**52d**	(1.25 ± 0.08) × 10^8^	(2.07 ± 0.06) × 10^8^	(4.46 ± 0.05) × 10^8^	(1.70 ± 0.01) × 10^8^	(3.06 ± 0.01) × 10^7^	(1.49 ± 0.00) × 10^7^	(8.33 ± 0.19) × 10^8^	(1.74 ± 0.08) × 10^8^	(3.27 ± 0.08) × 10^8^
**Fermentation days**	***LABS* (cfu/g)**								
	**H1**	**M1**	**L1**	**H4**	**M4**	**L4**	**H6**	**M6**	**L6**
**0d**	(5.13 ± 0.01) × 10^6^								
**10d**	(5.49 ± 0.82) × 10^6^	(2.87 ± 0.08) × 10^7^	(7.91 ± 0.82) × 10^6^	(4.64 ± 0.01) × 10^6^	(2.95 ± 0.16) × 10^7^	(3.50 ± 0.08) × 10^7^	(5.12 ± 0.82) × 10^6^	(1.65 ± 0.08) × 10^7^	(2.72 ± 0.08) × 10^7^
**21d**	(6.41 ± 0.82) × 10^6^	(8.56 ± 0.82) × 10^6^	(6.20 ± 0.82) × 10^6^	(6.17 ± 0.14) × 10^7^	(5.68 ± 0.08) × 10^7^	(6.09 ± 0.82) × 10^6^	(4.57 ± 0.01) × 10^6^	(9.06 ± 0.82) × 10^6^	(6.10 ± 0.82) × 10^6^
**52d**	(4.54 ± 0.01) × 10^6^	(6.12 ± 0.82) × 10^6^	(4.06 ± 0.87) × 10^6^	(3.38 ± 0.01) × 10^6^	(6.21 ± 0.16) × 10^7^	(4.32 ± 0.08) × 10^7^	(3.28 ± 0.01) × 10^6^	(4.44 ± 0.16) × 10^7^	(3.40 ± 0.01) × 10^6^
**Fermentation days**	***Yeasts* (cfu/g)**								
	**H1**	**M1**	**L1**	**H4**	**M4**	**L4**	**H6**	**M6**	**L6**
**0d**	(1.49 ± 0.08) × 10^5^								
**10d**	(1.94 ± 0.25) × 10^4^	(3.00 ± 0.00) × 10^2^	(1.65 ± 0.16) × 10^4^	(1.93 ± 0.16) × 10^4^	(0.00 ± 0.00) × 10^4^	(0.00 ± 0.00) × 10^4^	(1.90± 0.01) × 10^5^	(0.00 ± 0.00) × 10^4^	(0.00 ± 0.00) × 10^4^
**21d**	(6.90 ± 0.74) × 10^4^	(8.57 ± 0.48) × 10^4^	(1.01 ± 0.01) × 10^6^	(6.47 ± 0.08) × 10^5^	(0.00 ± 0.00) × 10^4^	(1.48 ± 0.33) × 10^4^	(2.69 ± 0.49) × 10^4^	(1.83 ± 0.02) × 10^6^	(8.70 ± 0.00) × 10^3^
**52d**	(2.88 ± 0.33) × 10^4^	(1.08 ± 0.01) × 10^4^	(2.70 ± 0.01) × 10^3^	(2.86 ± 0.33) × 10^4^	(0.00 ± 0.00) × 10^4^	(1.08 ± 0.01) × 10^3^	(2.43 ± 0.33) × 10^4^	(1.83 ± 0.33) × 10^4^	(6.70 ± 0.01) × 10^3^
**Fermentation days**	***Molds* (cfu/g)**								
	**H1**	**M1**	**L1**	**H4**	**M4**	**L4**	**H6**	**M6**	**L6**
**0d**	(6.90 ± 0.01) × 10^3^								
**10d**	(1.05 ± 0.08) × 10^6^	(8.41 ± 0.82) × 10^4^	(3.51 ± 0.08) × 10^5^	(8.92 ± 0.08) × 10^5^	(6.00 ± 0.00) × 10^3^	(6.00 ± 0.00) × 10^3^	(1.95 ± 0.37) × 10^5^	(1.44 ± 0.00) × 10^3^	(1.11 ± 0.00) × 10^3^
**21d**	(1.77 ± 0.41) × 10^5^	(3.69 ± 0.49) × 10^5^	(1.35 ± 0.08) × 10^6^	(1.53 ± 0.08) × 10^5^	(4.38 ± 0.00) × 10^4^	(6.12 ± 0.82) × 10^5^	(1.68 ± 0.45) × 10^5^	(6.60 ± 0.00) × 10^3^	(1.44 ± 0.82) × 10^5^
**52d**	(3.53 ± 0.08) × 10^6^	(3.87 ± 0.08) × 10^6^	(6.89 ± 0.25) × 10^6^	(7.08 ± 0.82) × 10^5^	(3.21 ± 0.82) × 10^6^	(5.91 ± 0.00) × 10^4^	(6.72 ± 0.00) × 10^4^	(1.56 ± 0.41) × 10^5^	(6.00 ± 0.00) × 10^3^

**Table 2:** The α diversity of three stages samples during fermentation.

Fermentation Days	ID	Bacteria			Fungi		
	Samples	Chao1	Shannon	Good Coverage	Chao1	Shannon	Good Coverage
10d	H1	76.75	3.13	0.999	741.96	5.05	0.978
	M1	72.13	2.31	0.999	114.14	2.58	0.998
	L1	336.79	4.91	0.997	139.44	2.19	0.998
	H4	67.60	2.53	0.999	381.84	6.03	0.997
	M4	806.30	8.48	0.991	87.00	4.59	1.000
	L4	444.94	6.21	0.996	485.66	3.96	0.990
	H6	82.75	1.23	0.997	286.06	2.47	0.995
	M6	488.02	5.58	0.989	210.94	3.29	0.995
	L6	796.48	6.91	0.989	156.00	2.37	0.998
21d	H1	54.50	2.01	0.999	539.37	3.71	0.987
	M1	239.11	2.42	0.996	532.06	1.87	0.989
	L1	106.17	2.86	0.998	402.40	2.88	0.991
	H4	323.27	3.74	0.990	467.58	5.97	0.993
	M4	296.14	2.38	0.990	428.18	2.60	0.992
	L4	55.00	1.72	0.999	512.76	2.88	0.988
	H6	71.60	2.96	0.999	439.09	2.14	0.986
	M6	146.00	3.39	0.997	19.33	1.39	0.999
	L6	71.33	2.70	0.999	312.13	4.04	0.996
52d	H1	213.37	3.23	0.999	466.74	3.76	0.988
	M1	156.38	2.00	0.999	435.82	4.09	0.986
	L1	223.64	3.39	0.999	354.97	2.81	0.999
	H4	205.58	2.79	0.999	255.33	2.18	0.998
	M4	371.84	3.99	0.998	360.46	1.28	0.989
	L4	150.86	1.94	1.000	183.42	2.08	0.999
	H6	317.41	3.66	0.999	89.53	1.64	0.999
	M6	217.33	3.09	1.000	248.70	2.95	0.998
	L6	313.73	2.47	0.996	97.39	2.21	0.998

## Data Availability

The datasets used in this study are available in online repositories. The repository names and corresponding accession numbers are as follows: NCBI (https://www.ncbi.nlm.nih.gov/), Accession Number: PRJNA1293933. NCBI (https://www.ncbi.nlm.nih.gov/), Accession Number: PRJNA1293917.

## Supplementary Materials

The following supporting information can be downloaded at https://www.mdpi.com/article/10.3390/biology14101324/s1, Figure S1: Sampling diagram, note: H1, M1, L1: top, middle and bottom of column 1; H4, M4, L4: the fourth column upper, middle and lower; H6, M6, L6: Top, middle and bottom of column 6; Figure S2: Microbial β-diversity across the three fermentation stages of *Daqu*.
