# Supplementary material for: Microbial, Physicochemical, and Flavor Interactions in High-Temperature Sauce-Flavor Daqu
Source: Biology (Basel). 2025 Sep 25;14(10):1324. doi: 10.3390/biology14101324 (PMC12561397; doi:10.3390/biology14101324)
Supplement: Supplementary file 1 [file biology-14-01324-s001.zip › biology-3814803-supplementary.pdf]

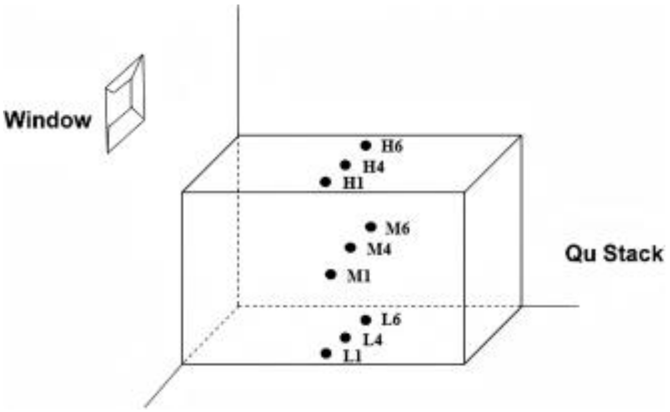

Figure S1. Sampling diagram.
Note: H1, M1, L1: top, middle and bottom of column 1; H4, M4, L4: the fourth column upper, middle and lower; H6, M6, L6: Top, middle and bottom of column 6.

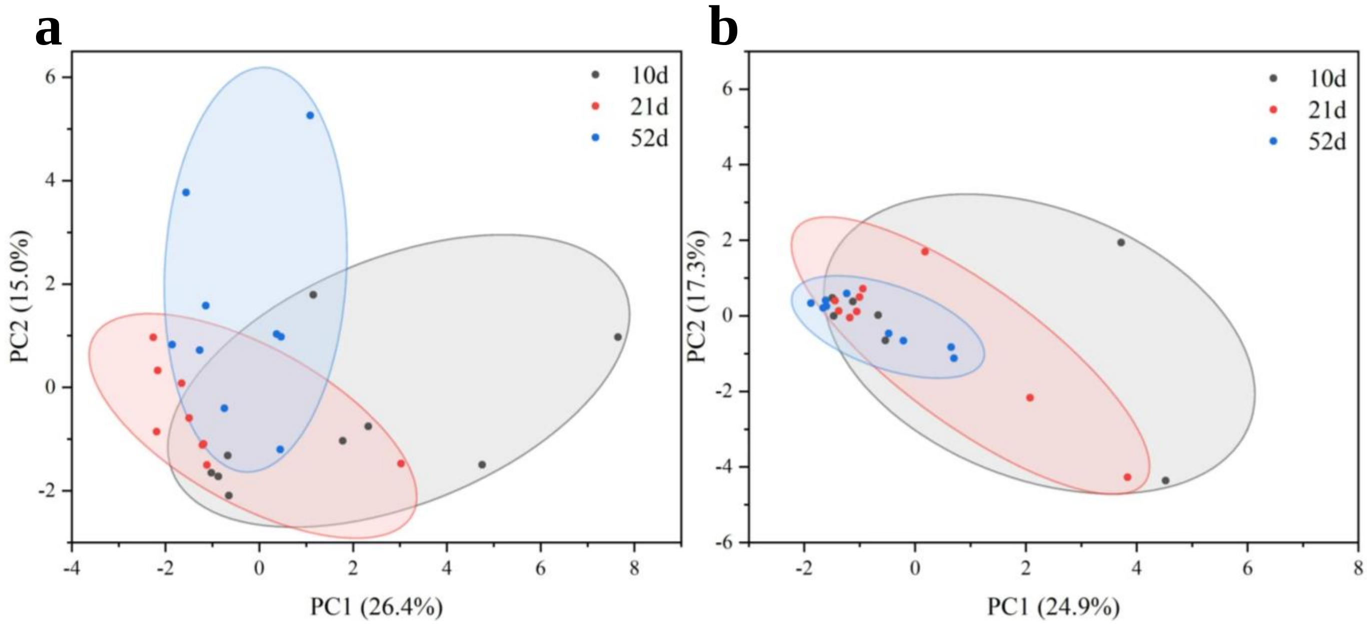

Figure S2. Microbial β-diversity across the three fermentation stages of Daqu: (a) bacterial community and (b) fungal community. β-diversity was assessed using Bray–Curtis distance with a 95% confidence level (R^2 = 0.8694, p = 0.0001).
